# Identification and characterization of the Cucurbitacins, a novel class of small-molecule inhibitors of Tropomyosin receptor kinase a

**DOI:** 10.1186/s12906-019-2709-z

**Published:** 2019-11-06

**Authors:** Yueling Zhong, Hong Xu, Yi Zhong, Xuemiao Zhang, Ting Zeng, Limei Li, Gaojie Xu, Minhui Li, Jin Liu, Tai Yang

**Affiliations:** 1 School of Pharmacy, Chengdu Medical College, No.783, Xindu Avenue, Xindu District, Chengdu, 610500 Chengdu Sichuan Province China; 2 School of Laboratory Medicine, Chengdu Medical College, Chengdu, China; 3 Center for Scientific Research, Chengdu Medical College, No.783, Xindu Avenue, Xindu District, Chengdu, 610500 Sichuan Province China

**Keywords:** Cucurbitacins, TrkA, Kinase, Inhibitors, Pruritus

## Abstract

**Background:**

NGF-TrkA is well known to play a key role in propagating and sustaining pruritogenic signals, which form the pathology of chronic pruritus. Inhibition of NGF-TrkA is a known strategy for the treatment of pruritus. In the present paper, we describe the identification, in vitro characterization, structure–activity analysis, and inhibitory evaluation of a novel TrkA inhibitory scaffold exemplified by Cucurbitacins (Cus).

**Methods:**

Cus were identified as TrkA inhibitors in a large-scale kinase library screen. To obtain structural models of Cus as TrkA inhibitors, AutoDock was used to explore their binding to TrkA. Furthermore, PC12 cell culture systems have been used to study the effects of Cus and traditional Chinese medicinal plants (Tian Gua Di and bitter gourd leaf) extracts on the kinase activity of TrkA.

**Results:**

Cus block the phosphorylation of TrkA on several tyrosine sites, including Tyr490, Tyr674/675, and Tyr785, and inhibit downstream Akt and MAPK phosphorylation in response to NGF in PC12 cell model systems. Furthermore, traditional Chinese medicinal plants (Tian Gua Di and bitter gourd leaf) containing Cu extracts were shown to inhibit the phosphorylation of TrkA and Akt. These data reveal mechanisms, at least partly, of the anti-pruritus bioactivity of Cus.

**Conclusion:**

Taken together, with the recent discovery of the important role of TrkA as a therapeutic target, Cus could be the basis for the design of improved TrkA kinase inhibitors, which could someday help treat pruritus.

## Background

**Fig. 1:**
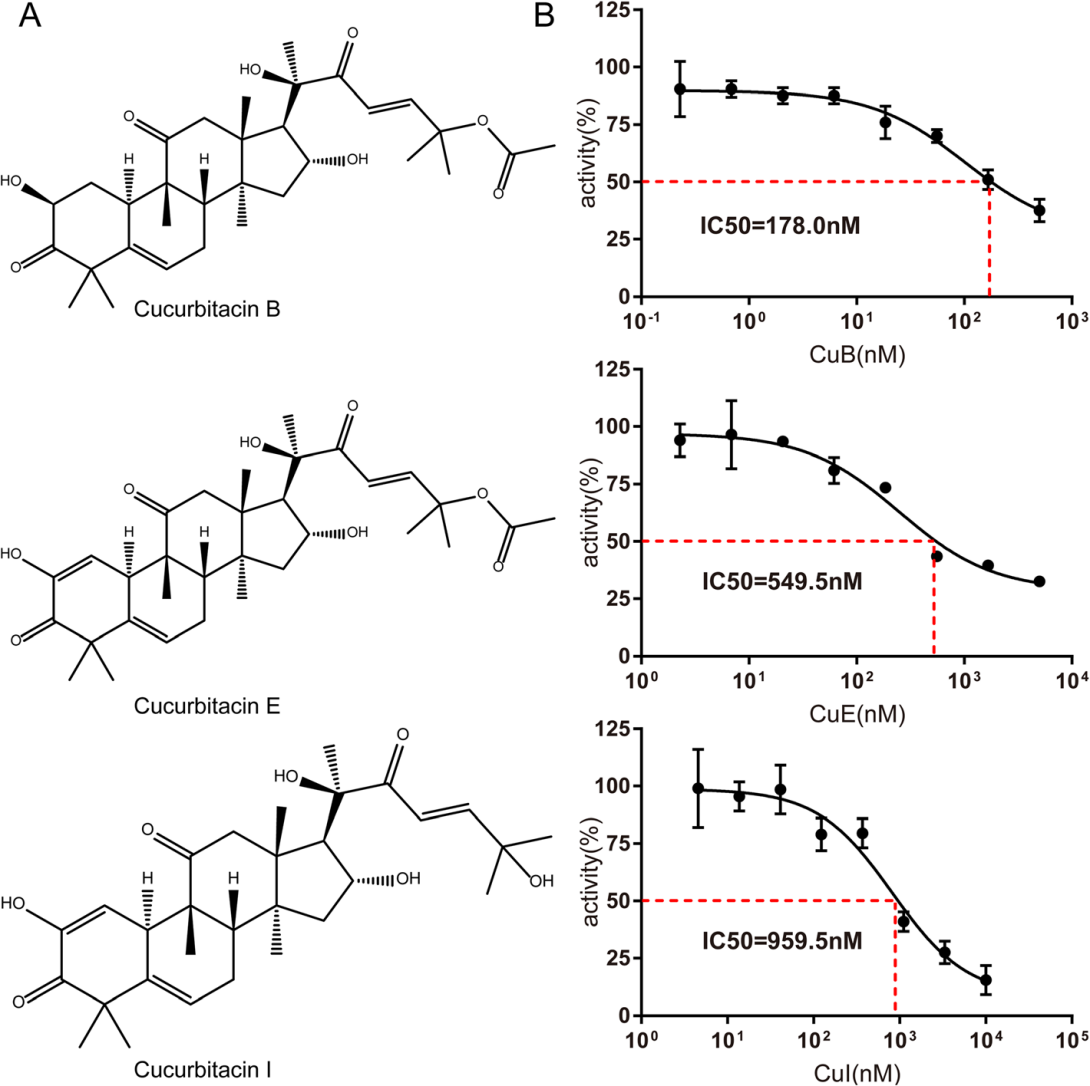
Cucurbitacins inhibit Tropomyosin receptor kinase A (TrkA) kinase activity at the cellular level**. (a**) Chemical structure of cucurbitacin B (CuB), CuE, and CuI. (**b**) TrkA activity in the presence or absence of increasing concentrations of CuB, CuE, and CuI. The results are presented as the percentage of kinase activity relative to the control (dimethyl sulfoxide), and the average of at least duplicate reactions, in which similar results were obtained

Cucurbitacins (Cus) are tetracyclic triterpenes, consisting of at least five cucurbitacin compounds, named Cucurbitacin B (CuB), Cucurbitacin D (CuD), Cucurbitacin E (CuE), Cucurbitacin I (CuI), and Cucurbitacin Q (CuQ). Among them, CuB, CuE, and CuI (Fig. 1a) have been widely studied. They are widely distributed in the Cucurbitaceae family and several other plants families, and have a range of biological and pharmacological activities. Cus were first used as an anti-hepatitis drug in the late 1970s in China [1–3]. Interest in Cus has grown in recent years, and various studies have demonstrated that Cu analogues have a wide range of biological activities, including, hepatoprotective, anti-cancer, and anti-inflammatory activities [4]. However, biological targets of the Cus or detailed molecular mechanisms underlying their biological activities remain elusive. Some studies have demonstrated that their anti-proliferative effects are associated with cell cycle arrest and apoptosis, mediated via inhibition of the Jak-STAT3 signaling pathway [5, 6], while others have indicated that Cus target cofilin, a critical mediator of actin dynamics, thereby disrupting actin assembly [7–9]. Our previous study showed that Cucurbitacin B (CuB) could inhibit phosphorylation of Aurora A in human multiple myeloma cells and induce their G2-M arrest and apoptosis [10]. Therefore, Cu exerts extensive pharmacological activity by regulating multiple cellular pathways, which promoted us to explore more potential targets of Cu.

High-throughput kinase screening provides a powerful platform to support profiling a compound against hundreds of pharmaceutical targets in a single screen [11]. Kinases catalyze the transfer of the γ-phosphate of ATP to a target substrate. A sequence of such phosphorylation events has been implicated in the modulation of various biological process, including proliferation, survival, apoptosis, metabolism, transcription, and differentiation [12]. Kinase phosphorylation is the basis of cell homeostasis, and aberrant kinase functions have been linked to malignant, inflammatory, and neurodegenerative disorders. Thus, human kinases are a major focal point of basic and drug discovery research. Many effective drugs act by hitting multiple targets rather than individual targets, which may be associated with superior efficacy and toxicity [13]. The kinase target of Cu has not yet been completely characterized, so a high-throughput kinase screen was carried out. In this study, we used kinase screening approaches to identify tropomyosin receptor kinase A (TrkA) as a target of Cu.

## Methods

### Reagents, cells and cell culture

The PC12 cells used in the present study were obtained from the American Type Culture Collection (ATCC, Rockville, MD, USA). The cells were maintained in Dulbecco’s modified Eagle’s medium (DMEM, Gibco, Gaithersburg, MD, USA) containing 10% (v/v) fetal bovine serum (FBS) and 5% horse serum, supplemented with penicillin/streptomycin (all from Gibco). Cus were obtained from Sigma-Aldrich (St. Louis, MO, USA) and were dissolved in dimethyl sulfoxide (DMSO) at a stock concentration of 10 mM. The TrkA kinase inhibitor, GW441756, was purchased from Selleck Chemicals (Houston, TX, USA). The drugs were dissolved in DMSO to an initial stock concentration of 10 mM, and aliquots were made and stored at − 80 °C until use.

### Plant materials

The Gua Di (*Cucumis melo* fruit peduncles) were collected from Wulian County (35°45′0.76″N, 119°12′11.01″E, altitude 272 m), Rizhao City, Shandong Province, China. Bitter gourd (*Momordica charantia*) leaves were collected from Xindu District (30°49′35.10″N, 104°09′19.77″E, altitude 500 m), Chengdu City, Sichuan Province, China in July 2016. Voucher specimens (Voucher number- GD01, BG01) have been deposited in the School of Pharmacy, Chengdu Medical College, Chengdu, China. These plants were identified by Professor Dr. Yuhang chen, Department of Pharmacy, The Chengdu Medical College.

### Ethanol extraction

Gua Di and Bitter gourd leaves were separately pulverized into fine powder using a stainless-steel blender and mixed with ethanol in a 1:10 ratio (w/v). The material was then extracted by boiling in a flask under reflux twice (1 h per cycle). Then, the extracts were concentrated under vacuum in a rotary evaporator. The decoction was lyophilized and stored at 4 °C. The lyophilized powder was dissolved in DMSO and filtered through a 0.22 μm syringe filter to create a stock solution. GDE and BGLE denote Gua Di and Bitter gourd leaf ethanol extract, respectively. GDE and BGLE were diluted with culture medium to the final concentration indicated for each experiment.

### HPLC analysis

The HPLC analysis was performed on a Waters 2695 apparatus coupled to a Waters 2996 photodiode array detector, assisted by Waters Empower 2 software. A Kromasil 100-5C18 (5 μm, 250 × 4.6 mm) column was used for analytical HPLC (mobile phase, 70% methanol, 1 mL/min), with the absorbance detection at 230 nm.

### Primary kinase screening

All assays were carried out by the International Centre for Protein Kinase Profiling (http://www.kinase-screen.mrc.ac.uk/) as described previously [14]. Briefly, all determinations were performed at room temperature and were in a linear range with respect to time and enzyme concentration. Assays were performed for 30 min using Multidrop Micro reagent dispensers (Thermo Fisher Scientific, San José, CA, USA) in a 96-well format. The concentration of magnesium acetate in the assays was 10 mM and [γ-^33^P] ATP (∼800 cpm/pmol) was used at 5 μM for protein kinases. The assays were stopped by adding 5 μL of 0.5 M (3%) orthophosphoric acid, and were then harvested onto Whatman® P81 UNIFILTER® plates with a washing buffer containing 50 mM orthophosphoric acid.

### Protein kinase activity assays

The assays to determine the 50% inhibitory concentration (IC50) values for each enzyme were performed as described previously [15]. In brief, TrkA (5–20 mU diluted in 50 mM Tris, pH 7.5, 0.1 mM EGTA, 0.1% β-mercaptoethanol, 1 mg/mL bovine serum albumin was assayed against a substrate peptide (Poly Glu Tyr) in a final volume of 25.5 μL, containing 50 mM Tris, pH 7.5, 0.1 mM EGTA, 1 mg/mL substrate peptide, 10 mM magnesium acetate, and 0.02 mM [γ-^33^P] ATP (50–1000 cpm/pmole), and was incubated for 30 min at room temperature. The assays were stopped by adding 5 μL of 0.5 M orthophosphoric acid, and were then harvested onto plates with a wash buffer of 50 mM orthophosphoric acid. Controls for solvents and background phosphorylation levels were included in all assays, and the non-specific ^33^P incorporation obtained was subtracted from all values. IC50 values were generated from log dose-response curves using Prism 5 software (GraphPad Software Inc., San Diego, CA, USA).

### Docking calculations

TrkA (PDB code: 4PMM) was obtained from the Protein Data Bank. The molecular structures of all compounds were obtained from PubChem. All water atoms were removed, and hydrogen atoms were added to the structures using the pyMol. The nonbonded interactions were calculated using all atomic parameters of AutoDock based on the AMBER force field, since they best reproduced crystal ligand conformations. Implicit solvation parameters were added from the AutoDock Tools package. Ligand nonpolar hydrogen atoms were marked as atom type ‘X’. Docking grids for carbon, oxygen, and polar and nonpolar hydrogen atoms were calculated by AutoGrid, also part of AutoDock. Grid maps contained points for TrkA and all of the compounds to constrain them within the docking cavity. Docking cavities were detected with AutoDockTools, and the candidate cavity was chosen referencing the inhibitor in the crystal structure, 4PMM. The binding conformation was screened with the Genetic Algorithm with Local Search docking method. The docking results were analyzed using pyMol.

### Western blot analysis

PC12 cells (4 × 10^6^ cells) were serum-starved in DMEM in the presence of CuB, CuE, CuI, GDE, BGLE, and GW441756 for 8 h. NGF (PeproTech, Rocky Hill, NJ) was added to the culture medium at a concentration of 50 ng/mL. After 5 min, the cells were lysed, and the protein concentration of all cell lysate samples was measured using the bicinchoninic acid (BCA) protein assay kit (Thermo Fisher Scientific, San José, CA, USA). The cell lysates with equal amounts of protein were mixed with SDS loading buffer containing 10% SDS, 50 mM dithiothreitol, 312 mM Tris-HCl (pH = 6.8), and 50% glycerin, boiled for 5 min, and separated by SDS-PAGE electrophoresis. The proteins were then transferred to polyvinylidene difluoride membranes (Roche Company, Basel, Switzerland). The membranes were blocked in PBS containing 0.05% (v/v) Tween®20 and 5% (w/v) nonfat dry milk at room temperature for 1 h. The primary antibody incubations were performed in TBS with 0.1% Tween®20 (TBST) with 5% nonfat dry milk overnight. After washing, horseradish peroxidase (HRP)-linked secondary antibodies (1:4000 dilution) were added and incubated for 1 h. Subsequently, the membranes were washed and incubated in ECL detection reagent (Millipore, Schwalbach, Germany). The following primary antibodies were used: rabbit anti-Phospho-TrkA 490 (1:2000), anti-Phospho-TrkA 674/675 (1:2000), rabbit anti-Phospho-TrkA 785 (1:2000), rabbit anti-Phospho-Akt 473(1:2000), rabbit anti-TrkA (1:2000), rabbit anti-Akt (1:2000) and rabbit anti-GAPDH (1:4000). All antibodies were purchased from Cell Signaling Technology, Danvers, MA, USA.

### Statistical analysis

GraphPad Prism 5.0 software was used to analyze data statistics. The results are presented as means ± standard deviations.

## Results

### Cucurbitacins suppresses TrkA activity

In the present study, the kinase selectivity of CuB was assessed using a radioactivity-based enzymatic assay against a kinase panel (Dundee profiling) [16]. CuB inhibited the kinase activity of TrkA by greater than 90% at 500 nM (Table 1), with an IC50 value of 178 nM (Fig. 1b), indicating that CuB is a potent inhibitor of TrkA. Then, we tested the ability of other Cus, CuE and CuI, to inhibit the activity of TrkA. As expected, both CuE and CuI significantly inhibited the phosphorylation of TrkA with an IC50 of 549.5 and 959.5 nM, respectively. These screening data provide the first direct evidence of the potential utility of Cus as novel TrkA inhibitors, However, these studies do not explain how Cus binds to the TrkA kinase. Thus, a computational docking study was performed to elucidate the binding mode of the TrkA kinase and its inhibitors.

**Table 1:** Effect of cucurbitacin B (CuB) on protein kinase activities in a high-throughput screening

Kinase	Remaining activity(%)	Kinase	Remaining activity(%)	Kinase	Remaining activity(%)		Remaining activity(%)
TrkA	5.2±0.8	BRSK2	74.9±1.6	HIPK3	91.3±4.0	PHK	106.4±8.4
CAMK1	12.9±3.7	ERK5	75.1±10.6	PKD1	91.6± 11.2	IRAK4	107.5±9.8
AMPK	22.6±1.5	MKK1	75.7±3.8	TIE2	92.2±2.5	p38d MAPK	108.1±21.4
Aurora B	23.2±2.8	IKKb	76.5±18.1	BRSK1	93.3±2.0	CK2	108.3±32.0
YES1	29.8±3.7	MAPK-K3	77.2±1.4	MNK1	94.2±15.4	ERK2	108.5±14.9
TSSK1	33.8±5.0	MLK3	77.4±13.5	ROCK 2	94.8±3.4	DAPK1	108.5±18.5
PIM3	40.6±5.2	SmMLCK	77.4±10.9	PKBa	94.9±14.1	PRK2	108.7±14.7
PAK4	42.3±4.6	CK1γ2	77.6±21.0	JNK2	95.3±1.0	PKA	109.2±15.3
PKCa	43.9±2.4	CLK2	78.5±5.5	TAK1	95.5±18.3	DDR2	109.4±7.8
HER4	46.2±15.1	MELK	79.5±14.7	STK33	96.3±7.0	MST2	109.6±30.2
DYRK1A	47.4±4.9	CSK	80.9±5.6	WNK1	97.0±1.4	OSR1	109.6±2.0
PKCγ	50.1±8.0	GCK	81.2±11.9	CDK9	97.4±14.2	JAK2	109.7±8.0
PAK2	53.1±5.5	EPH-B3	81.8±2.8	ULK2	98.6±4.1	MINK1	112.6±15.4
ERK8	54.3±2.7	MSK1	82.7±6.8	PDGFRA	99.2±8.5	NEK6	113.0±24.7
EF2K	55.1±1.2	PINK	83.2±9.7	CK1δ	99.3±28.6	MEKK1	114.2±13.1
CHK2	56.2±1.9	MAP4K3	83.9±22.2	IRAK1	99.5±2.4	NEK2a	114.4±11.3
MKK2	57.0±6.9	IR	84.1±5.7	SRPK1	99.6±13.2	TGFBR1	116.0±26.8
NUAK1	58.5±11.6	SIK2	84.5±12.6	RSK1	99.7±5.0	MNK2	116.8±19.1
PIM1	59.5±13.7	PDK1	84.6±9.2	TTBK2	100.3±11.8	EPH-A2	118.8±15.6
SYK	61.3±5.8	SGK1	84.9±4.2	ASK1	100.5±17.0	SIK3	120.9±26.3
PAK5	62.3±6.9	MAPK-K2	86.6±9.9	ERK1	101.6±4.4	TESK1	121.0±11.0
FGF-R1	62.3±7.4	TAO1	87.0±1.7	ULK1	102.0±11.2	BTK	122.0±45.3
VEG-FR	65.4±4.3	LKB1	87.0±11.2	IGF-1R	102.4±3.9	MKK6	125.9±6.4
HIPK2	65.6±1.6	p38a MAPK	87.3±13.8	TTBK1	102.5±14.3	PAK6	127.3±2.3
CDK2	66.7±3.6	Src	87.4±14.8	TLK1	102.5±25.8	EPH-A4	127.5±5.0
DYRK2	67.5±5.0	MARK1	87.6±11.4	MST4	102.8±6.4	MPSK1	127.9±43.9
MARK4	70.5±1.5	RIPK2	88.1±22.1	BRK	103.0±24.0	IRR	131.0±37.3
IKKe	71.2±12.5	PIM2	88.3±3.9	ZAP70	104.0±8.2	p38g MAPK	131.3±22.8
DYRK3	71.6±2.7	MAP4K5	88.9±15.4	MST3	104.3±10.8	p38b MAPK	139.7±0.8
TTK	71.9±2.6	MLK1	89.1±3.6	RSK2	105.0±11.4	PKCz	145.1±17.1
JNK3	72.2±13.0	JNK1	89.8±7.8	CAMKKb	105.7±2.0	Lck	157.4±11.1
PRAK	72.8±0.1	PLK1	89.8±13.2	MARK2	105.8±0.2	EPH-B4	160.4±40.7
GSK3b	73.2±8.2	PKBb	90.0±11.3	MARK3	105.9±5.5	EPH-B1	190.1±4.6
S6K1	73.7±27.0	EIF2AK3	90.7±9.1	ABL	106.2±23.9	CHK1	190.6±4.0
EPH-B2	74.3±6.4	TBK1	91.2±12.5	HIPK1	106.4±27.1		

Results presented indicate the remaining activity of each kinase (average of two duplicate determinations ± standard deviation) in the presence of 500 nM of CuB. Inhibition of tropomyosin receptor kinase A (TrkA) by CuB is indicated in red font

### Docking studies

**Fig. 2:**
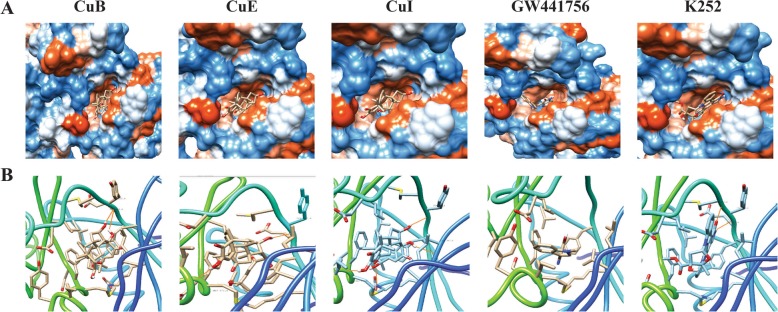
Two binding modes of Cucurbitacins in the tropomyosin receptor kinase A (TrkA) binding site. (**a**) The molecular surface of TrkA colored according to the electrostatic potential. (**b**) Closer view of the docking model of Cucurbitacins and TrkA. Hydrogen-bonding interactions are shown as orange lines and the hydrogen-bonding interactions of the amino acids stabilize the complex

To obtain structural models of Cus as TrkA inhibitors, AutoDock was used to explore their binding to TrkA. The model was generated using the coordinates of known TrkA crystal structures, particularly those of the complex with known TrkA inhibitor (PDB code 4PMM), without crystal water and coligands. Two reasonable binding modes of Cus in the pocket of TrkA were found (Fig. 2), in which Cus and positive controls (GW441756 and K252a) occupy approximately the same pocket (Cavity Volume 858 Å3), but are oriented differently. In both binding modes, the compounds, GW441756 and K252a, were the positive TrkA inhibitors. Our docking studies indicate that K252a interacts with TrkA through H-bonds with residues Met592, Tyr391, and Glu590, whereas no H-bond interactions were observed between GW441756 and TrkA. Similar results were obtained with the Cus and TrkA. As expected, CuB interacts with TrkA through H-bonds with residues Tyr591 and Glu590. CuE interacts with TrkA through H-bonds with residues Tyr591 and Met671. CuI interacts with TrkA through H-bonds with residues Tyr591, Ala542, and Met671. The hydrogen-bonding interactions of the amino acids stabilize the complex. Meanwhile, the binding affinity of the TrkA/ligand complexes was expressed in terms of docking scores. Docking scores of GW441756 and K252a against TrkA were − 140.49 and − 160.51, respectively. Similar results were obtained with the Cus and TrkA. The docking scores of CuB, CuE, and CuI against TrkA were − 138.11, − 153.78, and − 153.70, respectively. The more negative the docking score, the more favorable the interaction of the complex, so these results provide a theoretical possibility that Cus are potential TrkA inhibitors. This corroborates the kinase panel screening data, but it should be further supported by cellular TrkA pathway assays.

### Cucurbitacins inhibits NGF-mediated TrkA pathway in PC12 cells

**Fig. 3:**
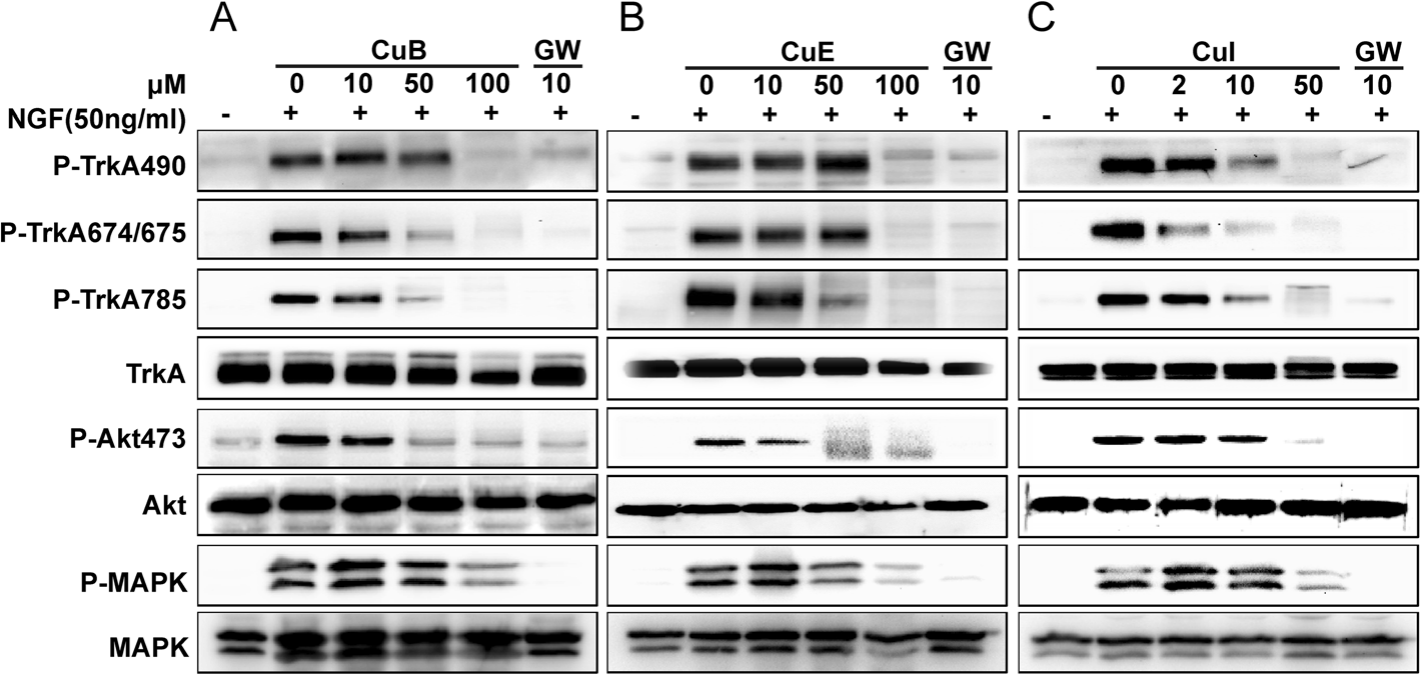
Cucurbitacins inhibit nerve growth factor (NGF)-mediated tropomyosin receptor kinase A (TrkA) pathway in PC12 cells. The inhibition of TrkA phosphorylation induced by cucurbitacin B (CuB), CuE, and CuI was assayed in the presence or absence of the indicated cucurbitacin concentrations, and the inhibitor GW441756 as a positive control. (**a**) CuB inhibited TrkA and downstream Akt phosphorylation in a concentration-dependent manner. (**b**) CuE inhibited TrkA and downstream Akt phosphorylation in a concentration-dependent manner. (**c**) CuI inhibited TrkA and downstream Akt phosphorylation in a concentration-dependent manner. The blot shown is representative of at least three experiments

In this regard, it is noteworthy that pheochromocytoma 12 (PC12) cells express TrkA receptor that mediates neurite outgrowth and anti-apoptotic signaling by binding to NGF [17]. Hence, PC12 cell culture systems have been used to study the effects of potential inhibitors on the kinase activity of TrkA. The PC12 cells were serum-starved for 6 h and pretreated with various concentrations of CuB, CuE, CuI before being stimulated with NGF (50 ng/mL). The results of western blot analysis clearly show that NGF stimulated TrkA phosphorylation on several tyrosine sites, including tyrosine 490, 674/675, and 785 (Fig. 3). This response was inhibited when the cells were pretreated with 10–100 μM CuB and CuE. Furthermore, CuI was more effective than CuB and CuE were in inhibiting TrkA phosphorylation, with 10 μM CuI significantly inhibiting TrkA phosphorylation (Fig. 3a). The inhibitory activity on TrkA phosphorylation induced by CuE seems to be the lowest, and CuE even induced the increased phosphorylation at TrkA490 site of TrkA at the higher concentration may due to CuE’s complicated biological mechanism (Fig. 3b). In PC12 cells, TrkA has been shown to promote sustained activation of the PI3K/Akt pathway, leading to increased cell survival [17]. Thus, upstream TrkA blockade is expected to inhibit downstream Akt phosphorylation. Indeed, we verified experimentally that Cus inhibit downstream Akt phosphorylation (Fig. 3). This provides further evidence that Cus are TrkA inhibitors.

### GDE and BGLE inhibits NGF-mediated TrkA pathway in PC12 cells

**Fig. 4:**
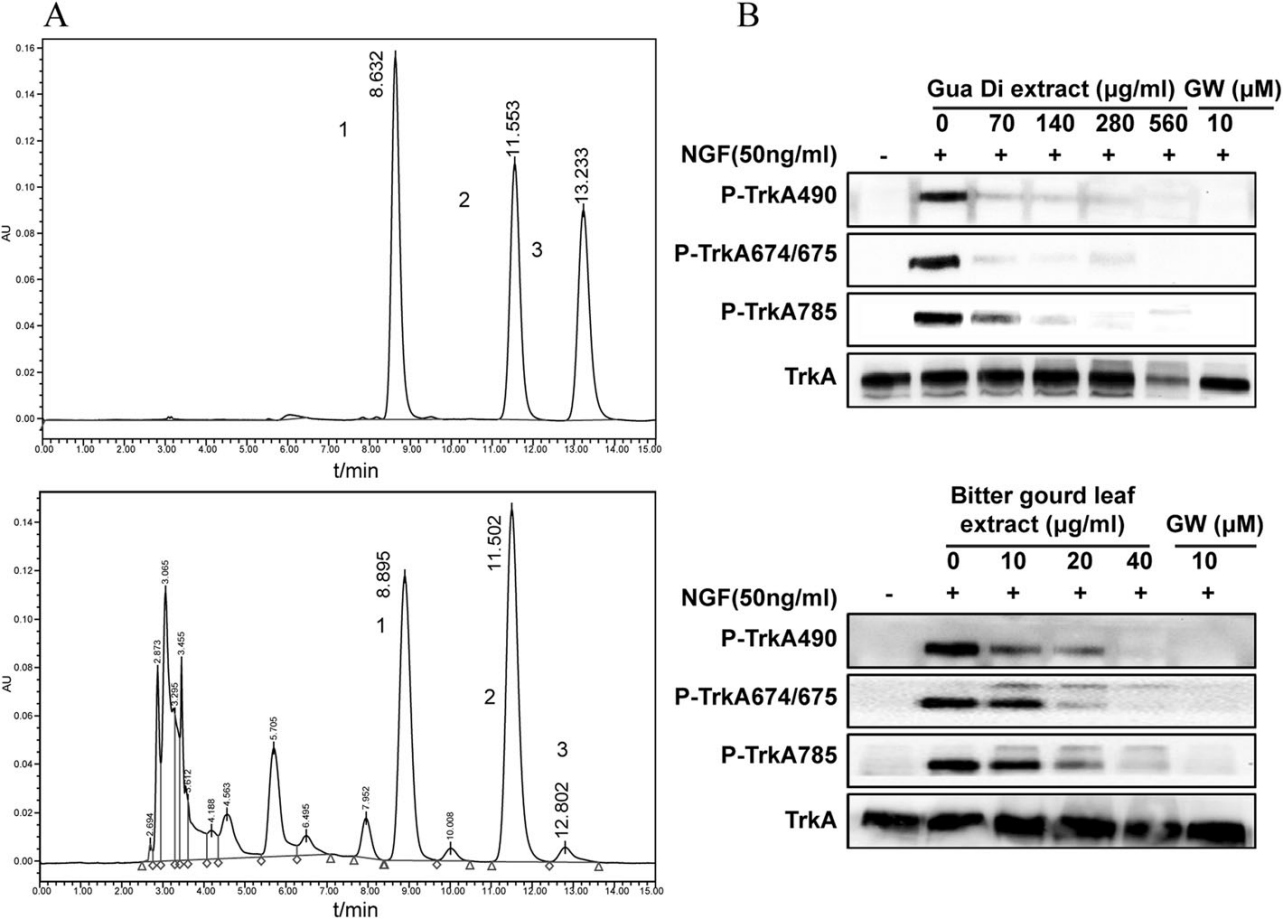
Gua Di extract (GDE) and Bitter gourd leaf extract (BGLE) inhibit nerve growth factor (NGF)-mediated tropomyosin receptor kinase A (TrkA) pathway in PC12 cells. (**a**) HPLC chromatogram of three standard compounds CuI (1), CuB (2) and CuE (3) respectively (upper panel); HPLC chromatograms of extracts from GDE detected at 230 nm (lower panel). Key to peak identities: cucurbitacin I (CuI) (1); CuB (2);CuE (3); (**b**) GDE (upper panel) and BGLE (lower panel) inhibited TrkA a phosphorylation in a concentration-dependent manner as shown by western blot

‘Gua Di’ (in Chinese), a traditional Chinese herb prescribed for liver disorders by native Chinese physicians, is rich in CuB and CuE [18]. It is noteworthy that Gua Di has also been used as a folk remedy to treat pruritus. TrkA aberrant activation plays important roles in pruritus and psoriatic plaque formation, and TrkA kinase inhibitor has been shown to be an effective and novel agent to treat patients with pruritus due to psoriasis [19]. To understand the mechanisms of anti-pruritus induced by Gua Di, the inhibitory effect of GDE on TrkA phosphorylation was evaluated. First, HPLC analysis of the original sample (Fig. 4a) indicates that the GDE contained CuI (retention time: 8.90 min), CuB (retention time: 11.50 min), and CuE (retention time: 12.80 min). The fractions corresponding to peaks 1, 2, and 3 contained CuI, CuB, and CuE, respectively, as confirmed via HPLC analysis using the Cu standards. Second, GDE caused a dose-dependent decrease in TrkA phosphorylation in starved PC12 cells; this effect was consequently associated with decreased phosphorylation of TrkA at Y490, Y674, Y675, and Y785. Interestingly, folk wisdom believes that bitter gourd (*M. charantia* L) leaf helps to prevent or counteract pruritus, and that the plants of the genera *Momordica* contain a special group of Cus [1]. Thus, we wanted to investigate if BGLE possessed the ability to inhibit TrkA activity, similar to that of the Cu derivatives. Our results show that BGLE does indeed inhibit TrkA phosphorylation from PC12 cells significantly in a dose-dependent manner (Fig. 4b).

## Discussion

Aberrant kinase function or regulation can contribute to the rise of many diseases [20]. While protein kinases have become therapy targets, only a small fraction of protein kinases are targeted by validated inhibitors [21]. High-throughput screening technology has become a key tool to screen several compounds against kinases quickly and effectively [21, 22]. In this study, we used kinase screening approaches to identify kinase targets of CuB. The strongest inhibitory activity was found against TrkA. TrkA, the surface transmembrane receptor tyrosine kinase for the neurotrophin, nerve growth factor (NGF), plays an important role in the pathogenesis of psoriasis and associated pruritus [23, 24]. Supporting this possibility is recent clinical evidence that TrkA kinase inhibition significantly reduced pruritus in patients with psoriasis [19]. Thus, TrkA, which plays a key role in the development and maintenance of cutaneous innervation, has emerged as a new therapeutic target for developing anti-pruritus treatments. To our knowledge, the present study is the first identification of Cus as TrkA kinase inhibitors that can be used to inhibit classical NGF/TrkA activity in cells. These data contribute to the use of Cu derivatives as lead compounds for the design and development of new agents against diseases with abnormal TrkA activation.

Unlike other protein kinase inhibitors currently in use, there have been relatively few reports of TrkA inhibitors. Most of these molecules share a similar structure with staurosporine. For example, both CEP-701 and K252a are TrkA inhibitors that are structurally related to staurosporine. Thus, characterization of novel classes of potent and specific TrkA inhibitors is still a challenge. Due to their important therapeutic promise, significant efforts to identify novel TrkA inhibitors have been made in recent years. The natural product, wrightiadione, was discovered as a new template for the development of TrkA inhibitors. The wrightiadione derivative, 2 h, showed a potent inhibitory activity (IC50 = 6.6 μM) toward TrkA at the molecular level [25]. In the present study, we describe Cu as a novel, cell-permeable inhibitor class of TrkA, which specifically inhibits TrkA with an IC50 value of 178–959.5 nM. CuI is the most potent, inhibiting NGF-mediated TrkA phosphorylation at the cellular level at 10 μM. Due to their potency as TrkA inhibitors, Cus provide an attractive platform to design derivatives with enhanced inhibitory activity toward TrkA.

The discovery of artemisinin came from an intensive search for plant natural products representing the wisdom of Chinese medicine [26, 27]. The application of cucurbitacin against chronic hepatitis is another successful example of Chinese medicine’s influence on innovative drug discovery. The Ben Cao Gang Mu, published in 1596, describes the characteristics and applications of all the medicines listed, and it records the usage of Gua Di for treating icterus..In addition, Gua Di was described by Wang Tao in his treatise “Wai Tai Mi Yao” (Chinese Tang Dynasty). This prescription is well known for the treatment of skin-related diseases such as psoriasis, pruritus, and dermatitis. The fact that Cus inhibit TrkA provides a possible molecular mechanism to explain this effect.

There is evidence that the NGF-TrkA pathway plays a key role in the pathogenesis of psoriasis and associated pruritus. Human mast cells express functional TrkA, are a source of NGF, and NGF-induced histamine secretion from mast cells induces itch or pruritus [28, 29]. Keratinocytes also synthesize and secret NGF, and keratinocytes derived from psoriatic plaques synthesize higher levels of NGF compared to that of normal subjects [30, 31]. Abnormal NGF production induces pruritus through the sensitization of the peripheral sensory nerve terminals in the skin, the majority of which express TrkA. Thus, inhibition of the NGF-TrkA pathway is an effective strategy for treating pruritus.

## Conclusion

In conclusion, Cus block the phosphorylation of TrkA on several tyrosine sites, including Tyr490, Tyr674/675, and Tyr785, and inhibit downstream Akt and MAPK phosphorylation in response to NGF in PC12 cell model systems. Furthermore, traditional Chinese medicinal plants (Tian Gua Di and bitter gourd leaf) containing Cu extracts were shown to inhibit the phosphorylation of TrkA and Akt. These data reveal mechanisms, at least partly, of the anti-pruritus bioactivity of Cus. Considering the development of this TrkA inhibitor, together with the recent discovery about the important role of TrkA as a therapeutic target, Cu could act as a precursor for the development of improved TrkA kinase inhibitors with enhanced activity, which might one day contribute to the treatment of pruritus due to psoriasis.

## Data Availability

The datasets and study materials will be available from the corresponding author on request.

## Abbreviations

Cu: Cucurbitacin
NGF: Nerve growth factor
TrkA: Tropomyosin receptor kinase A
